# Wall shear stress in hypertensive patients is associated with carotid vascular deformation assessed by speckle tracking strain imaging

**DOI:** 10.1186/2056-5909-20-10

**Published:** 2014-09-25

**Authors:** Joung Wook Yang, Kyoung Im Cho, Je Hun Kim, Soo Young Kim, Cheol Su Kim, Ga In You, Jin Young Lee, Seon Yoon Choi, Sea Won Lee, Hyun Soo Kim, Jung Ho Heo, Tae Joon Cha, Jae Woo Lee

**Affiliations:** Division of Cardiology, Department of Internal Medicine, Kosin University College of Medicine, 262 Gamcheon-ro, Seo-gu, Busan 602-702 Korea

**Keywords:** Hypertension, Shear stress, Carotid arteries, Strain

## Abstract

**Introduction:**

Wall shear stress (WSS) is critically important in both vascular remodeling and atherosclerosis. Carotid intima-media thickness (IMT) and deformation parameters have been used as relevant indicators of carotid atherosclerosis. This study aimed to investigate the relationships between hemodynamic parameters in the common carotid artery (CCA) and the severity of carotid atherosclerosis in untreated hypertensive patients.

**Methods:**

Carotid artery ultrasound was performed in 100 untreated hypertensive patients. Morphologic and hemodynamic parameters of the CCA, including peak and mean WSS, global circumferential strain, peak posterior radial strain assessed by two-dimensional speckle tracking method, and IMT, were measured.

**Results:**

In patients with hypertension, there were significant correlations between carotid strain parameters and peak/mean WSS. Stepwise multiple regression analysis for carotid strain parameters after adjustment for age, carotid IMT, and brachial pulse wave velocity showed that peak WSS was an independent determinant of peak posterior radial strain (*p* = 0.009) and global circumferential strain (*p* = 0.002).

**Conclusions:**

These findings indicate that local shear stress is associated with carotid vascular deformation, which could be an underlying mechanism for the progression of atherosclerosis.

**Electronic supplementary material:**

The online version of this article (doi:10.1186/2056-5909-20-10) contains supplementary material, which is available to authorized users.

## Introduction

Hypertension is a known major risk factor for atherosclerosis, which is the leading cause of death in developed countries [1]. Although atherosclerosis is a systemic, multifactorial disease, the process of atherosclerosis preferentially affects the outer edges of vessel bifurcations [2]. The focal nature of atherosclerotic processes may be due to local hemodynamic factors [3], such as shear stress.

Wall shear stress (WSS) is a lateral biomechanical force that is determined by blood flow, vessel geometry, and fluid viscosity [3] and is directly related to vascular functioning including the regulation of vascular caliber and inhibition proliferation, thrombosis, and inflammation of the vessel wall [4]. Thus, WSS is atheroprotective and critically important in both vascular remodeling and atherosclerosis. The ratio between the maximum velocity at the center of the artery and vessel radius is a common approximation of WSS [5]. Lower WSS is known to be associated with the development of atherosclerotic plaques, as was observed in carotid arteries in subjects with risk factors for atherosclerosis [6–8]. Among patients with unilateral carotid atherosclerosis, shear stress is lower in carotid arteries with plaques than in contralateral plaque-free arteries [7].

Carotid intima-media thickness (IMT) and deformation parameters have been used as relevant indicators of carotid atherosclerosis [9–12]. Arterial wall stiffness estimated by speckle tracking imaging may present a different aspect of atherosclerosis from these conventional parameters of atherosclerosis. The different mechanism between functional and structural alternations of vessel walls secondary to atherosclerosis has been described in previous studies [11, 12]. These observations may indicate the importance of an integrated approach to assess the severity of atherosclerosis. This study aimed to investigate the relationships between hemodynamic parameters in the common carotid artery (CCA) and the severity of carotid atherosclerosis in untreated hypertensive patients. Because various anti-hypertensive drugs have vasodilating properties, which may impact the hemodynamic parameters of the CCA, we included patients with newly diagnosed or untreated hypertension.

## Methods

### Study population

One hundred patients with untreated or newly diagnosed essential hypertension were enrolled in this cross-sectional study. Exclusion criteria for both groups were diabetes mellitus, pregnancy, renal failure, anemia, chronic hepatopathy, nephrotic syndrome, hypothyroidism, and history of cardiovascular disease (hypertension, coronary artery disease, history of coronary angiography, or congestive heart failure). All subjects using lipid-lowering drugs such as statins or fibrates (in the previous 3 months) were also excluded. Normal sinus rhythm with a rate of 60 to 100 beats/min on a resting electrocardiogram was also required. Patients were asked if they were current smokers or nonsmokers. Blood pressure was measured on the right arm, after 5 min of rest, while in a sitting position. Diagnostic criteria of hypertension were assessed according to the Seventh Report of the Joint National Committee on Prevention, Detection, Evaluation, and Treatment of High Blood Pressure [13]. This study was approved by the Kosin University institutional review board, and informed consent was obtained from all participants.

### Common carotid artery ultrasound

We scanned bilateral CCA, carotid bifurcations, and the origins of the internal carotid arteries in longitudinal and transverse planes using Vivid 7 (GE Medical Systems, Milwaukee, WI, USA) equipped with a 14-MHz linear array scanner capable of providing conventional two-dimensional ultrasound images and strain images. The reader was the same throughout the study and was blinded to the subject investigated. All subjects were examined in a supine position, with the neck extended and the chin facing the opposite side. After placing the regions of interest in the far wall of the CCA, mean IMT was estimated in a region free of atherosclerotic plaques with the use of an automatic tracking system [14]. IMT was considered normal if it was less than 0.9 mm, and a plaque was defined as a focal structure encroaching into the arterial lumen by at least 0.5 mm or 50% of the surrounding IMT value or if it had a thickness >1.5 mm as measured from the media-adventitia interface to the intima-lumen interface [15, 16]. Ultrasound measurements were performed in the CCA 1 to 2 cm proximal to the bifurcation. The distal segments of the CCA were recorded digitally for further analysis. Blood flow velocity was detected with the sample volume placed in the center of the CCA. Peak systolic (Vpeak), end-diastolic, and mean (Vmean) velocities were recorded for at least three cardiac cycles. The systolic internal diameter of the CCA (SD) and the diastolic CCA diameter (DD) were acquired at the peak T and R waves for calculating carotid hemodynamic parameters. WSS was calculated using the Poiseuillian parabolic model of velocity distribution across the arterial lumen based on the assumption of laminar blood flow, according to the following formulae [17, 18]:

PeakWSS=8×μ×Vpeak/SD

MeanWSS=8×μ×Vmean/DD

where *μ* is blood viscosity, assumed to be 0.035 dyn × s/cm^2^ in patients with normal hematocrit [18]. The inter-observer coefficients of variation for peak and mean WSS were between 6% and 8%, respectively, which are similar to a previous study [19].

### Speckle tracking strain imaging in the carotid artery

An optimal short-axis image of the CCA during a breath hold at end-expiration was obtained and digitally stored for off-line analysis. Three heartbeats were collected from each view, and a single selected cycle was analyzed off-line with an EchoPAC Dimension system (General Electric, Horten, Norway). During systole, circumferential strain assumes positive values due to stretching or expansion of the vessel wall whereas radial strain becomes negative as a result of the compression of the vessel wall. As we previously reported [20], regions of interest (ROIs) with computation areas of 1 × 1 mm were placed in the intima-media complex from the short-axis view of the CCA, and circumferential peak systolic strain (%) was measured as an average of the whole, circular ROI giving respective ‘global’ strain. ‘Global’ values for radial strain could not be calculated due to limitations of the EchoPAC software, and consequently, radial peak systolic strain was only obtained ‘regionally’ from a discrete point (20 × 20 pixels) located in the far wall of the vessel. Inter-observer and intra-observer variabilities for strain were 16% and 18%, respectively.

### Measurement of arterial stiffness

Arterial stiffness was assessed by measuring brachial ankle pulse wave velocities (baPWVs) using an automatic waveform analyzer (VP-1000; Colin Co., Komaki, Japan). For measuring baPWV, pulse waves obtained from the brachial and tibial arteries were recorded simultaneously, and the transmission time, which was defined as the time interval between the initial increase in brachial and tibial waveforms, was determined. The transmission distance from the arm to each ankle was calculated according to body height. The baPWV was automatically computed as the transmission distance divided by the transmission time.

### Laboratory evaluation

A venous blood sample was collected on the day of examination after an overnight fast of at least 8 h. All hypertensive patients and controls were fasting for at least 12 h before blood tests at the beginning of the study. The following parameters were obtained by standard techniques on the day of examination: total cholesterol, low-density lipoprotein cholesterol, high-density lipoprotein cholesterol, triglycerides, and high-sensitivity C-reactive protein.

### Statistical analysis

Statistical analysis was performed with the statistical program SPSS ver. 12.0 (SPSS Inc., Chicago, IL, USA). Results are presented as the mean ± standard deviation or percentage. Comparisons were performed between patients with hypertension and controls using the Student *t*-test for quantitative variables and the chi-square or Fisher’s exact test for qualitative variables. Correlations between variables were made by calculating the correlation coefficient through Pearson correlation tests. Statistical significance was noted at a *p* value less than 0.05.

## Results

**Figure 1:**
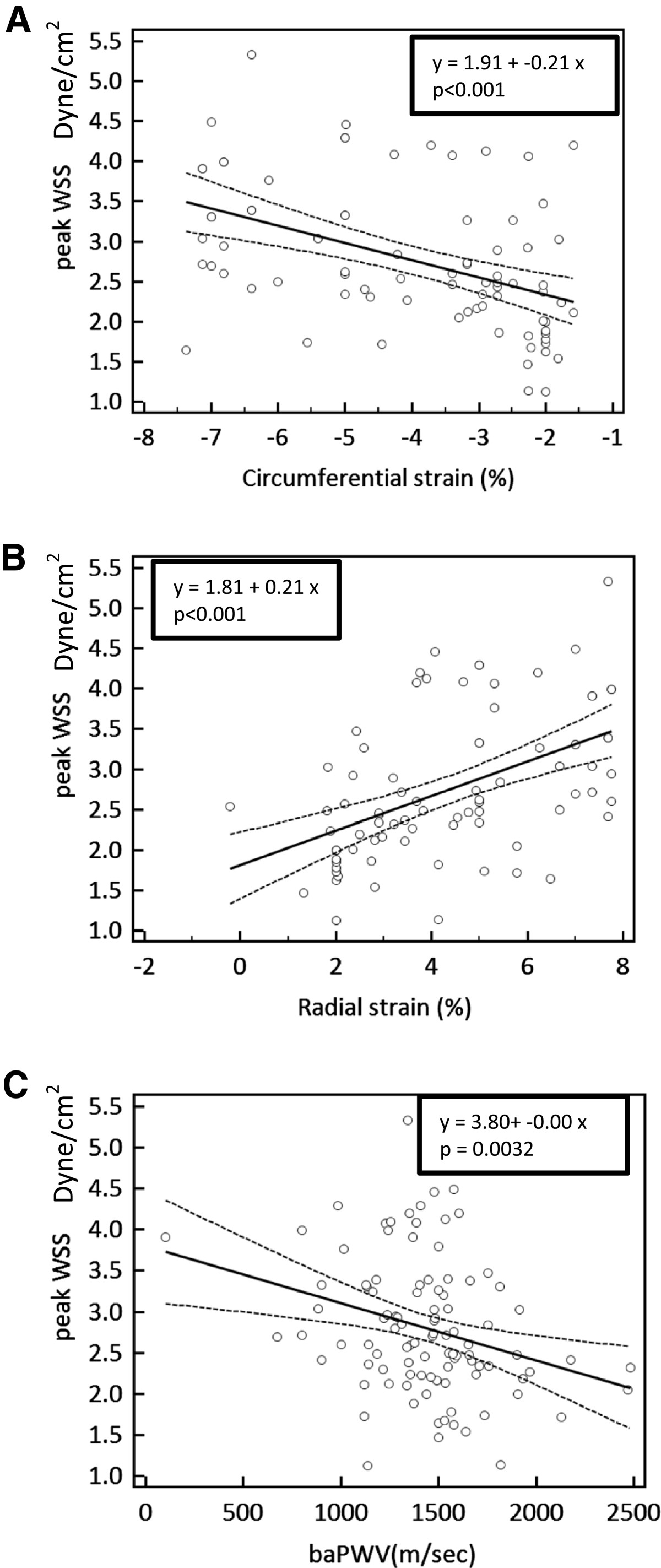
**Regression analysis between peak wall shear stress (WSS) and carotid deformation parameters assessed by speckle tracking echocardiography. (A)** Global circumferential strain and **(B)** peak posterior radial stain showed a significant positive correlation with peak WSS. **(C)** Brachial ankle pulse wave velocity (baPWV) showed a significant negative correlation with peak WSS.

Baseline clinical characteristics and parameters of the carotid artery in hypertensive patients are listed in Tables 1 and 2. In subjects with hypertension, lower carotid WSS was accompanied by increased age (*r* = -0.205, *p* = 0.034), higher carotid mean IMT (*r* = -0.236, *p* = 0.012), higher baPWV (*r* = -0.236, *p* = 0.012), and reduced peak posterior radial strain and global circumferential strain (*r* = -0.449, *p* < 0.001 and *r* = 0.492, *p* = 0.012, respectively) (Table 3). Systolic blood pressure showed no significant effect on global circumferential or peak posterior radial strain. On univariate regression analysis, peak WSS significantly affected the baPWV, global circumferential strain, and peak posterior radial strain (Figure 1). Multiple regression analysis was performed to evaluate contributing factors of decreased carotid deformation parameters in hypertensive subjects (Table 4). After adjustment for age, carotid IMT, and baPWV, peak WSS was an independent determinant of global circumferential strain (*p* = 0.009) and peak posterior radial strain (*p* = 0.002).

**Table 1:** **Baseline clinical characteristics of hypertensive patients**

Characteristic	Hypertensives (*n* = 100)
Age (years)	60.1 ± 9.8
Body mass index (kg/m^2^)	24.6 ± 3.0
Systolic BP (mmHg)	148.3 ± 14.9
Diastolic BP (mmHg)	92.1 ± 13.3
Hematocrit (%)	44.3 ± 8.7
Total cholesterol (mg/dL)	174.9 ± 46.8
Low-density lipoprotein cholesterol (mg/dL)	110.4 ± 40.9
High-density lipoprotein cholesterol (mg/dL)	50.5 ± 12.6
Triglycerides (mg/dL)	147.9 ± 86.6
Free T4 (pmol/L)	1.17 ± 0.16
Thyroid-stimulating hormone (mIU/L)	1.70 ± 1.43
High-sensitivity C-reactive protein (mg/L)	4.12 ± 5.98
Brachial ankle pulse wave velocity (m/s)	1,511.4 ± 323.3

Values are presented as mean ± standard deviation.

*BP* blood pressure.

**Table 2:** **Comparison of parameters of carotid arterial stiffness, hemodynamics, and atherosclerosis between normotensive controls and hypertensive patients**

Variable	Hypertensives (*n* = 100)
Left ventricular outflow tract deceleration time (ms)	175.9 ± 19.3
CCA mean intima-media thickness (mm)	0.90 ± 0.27
CCA systolic diameter (mm)	7.2 ± 0.8
CCA diastolic diameter (mm)	6.4 ± 0.7
Peak systolic velocity (cm/s)	60.6 ± 13.7
End diastolic velocity (cm/s)	13.8 ± 3.3
Mean velocity (cm/s)	37.2 ± 7.8
Peak WSS (dyn/cm^2^)	2.63 ± 0.75
Mean WSS (dyn/cm^2^)	1.38 ± 0.67
Pulsatility index	0.31 ± 0.03
Resistive index	0.76 ± 0.05
Global circumferential strain (%)	3.74 ± 1.78
Peak posterior radial strain (%)	-3.28 ± 1.52

Values are presented as mean ± standard deviation.

*CCA* common carotid artery, *WSS* wall shear stress.

**Table 3:** **Correlation coefficients between carotid parameters in the hypertensive group (** ***n*** **= 100)**

Variable	Peak wall shear stress		Carotid mean IMT	
	*r*	*p*value	*r*	*p*value
Age	-0.205	0.034	0.433	<0.001
Systolic blood pressure	-0.032	0.744	-0.005	0.954
Total cholesterol	0.050	0.632	-0.080	0.445
High-sensitivity C-reactive protein	-0.086	0.495	0.037	0.769
Pulsatility index	0.186	0.048	0.204	0.029
Resistive index	0.189	0.045	0.208	0.026
Brachial ankle pulse wave velocity	-0.292	0.003	0.265	0.007
Global circumferential strain	0.492	<0.001	-0.296	0.008
Peak posterior radial strain	-0.449	<0.001	0.236	0.033
Carotid mean IMT	-0.236	0.012	1	

*IMT* intima-media thickness.

**Table 4:** **Multiple regression analysis of factors affecting carotid deformation parameters in hypertensive subjects**

Variable	*B*	*p*value	95% confidence interval of *B*
Global circumferential strain (adjusted *R*^2^ = 0.204)			
Age	-0.005	0.968	-0.047 to 0.045
baPWV	-0.178	0.139	-0.002 to 0.000
Carotid mean IMT	0.032	0.794	-1.671 to 2.175
Peak WSS	0.424	0.001	0.414 to 1.412
Posterior radial strain (adjusted *R*^2^ = 0.204)			
Age	-0.014	0.912	-0.046 to 0.041
baPWV	0.274	0.023	0.000 to 0.003
Carotid mean IMT	0.003	0.977	-1.793 to 1.845
Peak WSS	-0.366	0.002	-1.224 to -0.280

*IMT* intima-media thickness, *baPWV* brachial ankle pulse wave velocity, *WSS* wall shear stress.

## Discussion

Our data demonstrate that local shear stress is associated with carotid vascular deformation in hypertensive patients, which could be an underlying mechanism for the progression of atherosclerosis.

The parallel frictional drag force of shear stress is one of the important blood flow-induced mechanical stresses acting on the vessel wall. WSS is proportional to the product of blood viscosity and spatial gradient of blood velocity at the wall. It is well established that WSS is an important determinant of endothelial cell function, and there is increasing evidence that low WSS expresses an atherogenic endothelial gene profile [3, 4, 21]. Moreover, WSS regulates arterial diameter by modifying the release of vasoactive mediators by endothelial cells [4, 22]. Therefore, mechanical shear stress plays an important role in hypertension both directly and indirectly via the release of bioactive molecules [23]. Because of the lack of techniques to assess WSS *in vivo*, WSS has been calculated according to Poiseuille’s law using the recorded velocity profiles and whole blood viscosity in large arteries [5]. In our study, the decrease in WSS of the CCA was due to the reduced blood flow velocity and luminal enlargement. A previous study showed that carotid arterial inter-adventitial distance (diameter) is an indicator of the damaging effects of age and atherosclerosis [24] and is most likely an adaptive phenomenon [25]. Our results are in agreement with previous observations that showed that hypertension is associated with carotid artery remodeling [26, 27]. Because high blood pressure exerts a fatiguing effect on the elements of the arterial wall such as elastin and collagen, degenerative changes and inter-adventitial enlargement, as well as alteration of blood flow velocity, may result [28, 29]. As expected, carotid mean IMT was higher in our hypertensive group than in normotensive controls, which reflects either intimal thickening, thickening of the medial layer, or a combination of both.

The unique finding in our study is that a strong inverse correlation between WSS and carotid deformation parameters indicated speckle tracking strain as well as baPWV, which indicates a low WSS effect on arterial stiffness. This supports our hypothesis that altered vascular stiffness is associated with hemodynamic vascular function, which might promote atherosclerosis. During left ventricular ejection, the systolic cardiac forces result in the exertion of hemodynamic forces on the carotid artery wall. The endothelial cells on the luminal side of the carotid artery sense the pressure pulse and a tangential stress exerted by the flowing blood. The difference between diastolic and systolic BP induces the systolic increase in diameter (radial strain) and cross-sectional area relative to the end-diastolic level (circumferential wall strain). The tangential stress exerted by the flowing blood is known as WSS, and the radial and circumferential thickening of the carotid arterial wall can be assessed by speckle tracking imaging-based strain parameters, as we previously reported [20]. Decreased elasticity of the arterial wall may be present even before the occurrence of any clinical symptoms or atherosclerotic plaques [30, 31]; therefore, evaluation of elastic properties of the arterial wall may provide efficient identification of individuals in the early stages of atherosclerotic disease. In our patients with hypertension, the decrease in WSS is explained by the increase in arterial diameter in order to reduce the loss of arterial compliance, which is connected with the reduced deformation of the carotid vascular wall. In a situation with low WSS, the resultant stagnation of blood permits increased uptake of atherogenic blood particles as a result of increased residence time [6]. WSS can also change the morphology and orientation of the endothelial cell layer [21]. Exposure of the arterial wall to a relatively low WSS may increase the vulnerability of such regions of the vessel to atherosclerosis [21]. Moreover, low WSS modulates the transcription of genes for nitric oxide and increased local production of mitogenic substances [32]. Consequently, the biomechanical force (low WSS) affects the vascular elastic properties (carotid wall deformation), promoting atherosclerosis.

This study has some limitations that should be considered. The first is related to the small number of patients and selection of the study population. Further study with a larger population should be undertaken to overcome this limitation. Second, the measurement of the WSS was based only on Poiseuille’s law, which is a common approximation of WSS [7, 33, 34]. However, when assuming a parabolic velocity profile, the underestimation of WSS might potentially lead to inaccuracy. However, even actual measurements of viscosity must not lead to real values of shear stress [35], and the use of an arbitrary value of blood viscosity would not change the statistical significance of the results [6, 36]. Finally, patients enrolled in our study would be at relatively earlier stages of hypertension because the inclusion criteria eliminated patients treated for hypertension, so the majority of the study cohort (82%) had stage I hypertension. Therefore, the prevalence of carotid plaques was not common (12%), and we did not perform subgroup analysis according to the presence of carotid plaques, which is the acute surrogate marker of carotid atherosclerosis. Nonetheless, this study highlighted that a reduced WSS independently affects reduced carotid deformation parameters even before the occurrence of any clinical symptoms or atherosclerotic plaques. Routine carotid ultrasonography is recommended in hypertensive patients with these independent predictors.

## Conclusions

It can be concluded from this study that local shear stress in hypertensive patients is associated with carotid vascular deformation, which could be an underlying mechanism for the progression of atherosclerosis.

## Authors’ original submitted files for images

Below are the links to the authors’ original submitted files for images. Authors’ original file for figure 1

## Abbreviations

WSS: Wall shear stress
IMT: Intima-media thickness
CCA: Common carotid artery
DM: Diabetes mellitus
US: Ultrasound
2D: Two-dimensional
Vpeak: Peak systolic velocity
Vmean: Mean velocity
SD: Systolic internal diameter
DD: Diastolic diameter
ROI: Regions of interest
baPWV: brachial ankle pulse wave velocity
HsCRP: High-sensitivity C-reactive protein
SD: Standard deviation
BP: Blood pressure.

## References

[1] Ezzati M, Lopez AD, Rodgers A, Vander Hoorn S, Murray CJ (2002). Comparative risk assessment collaborating group. Selected major risk factors and global and regional burden of disease. Lancet.

[2] Fox B, James K, Morgan B, Seed A (1982). Distribution of fatty and fibrous plaques in young human coronary arteries. Atherosclerosis.

[3] Asakura T, Karino T (1990). Flow patterns and spatial distribution of atherosclerotic lesions in human coronary arteries. Circ Res.

[4] Busse R, Fleming I (1998). Pulsatile stretch and shear stress: physical stimuli determining the production of endothelium-derived relaxing factors. J Vasc Res.

[5] Hoeks AP, Samijo SK, Brands PJ, Reneman RS (1995). Noninvasive determination of shear-rate distribution across the arterial lumen. Hypertension.

[6] Zarins CK, Giddens DP, Bharadvaj BK, Sottiurai VS, Mabon RF, Glagov S (1983). Carotid bifurcation atherosclerosis. Quantitative correlation of plaque localization with flow velocity profiles and wall shear stress. Circ Res.

[7] Gnasso A, Irace C, Carallo C, De Franceschi MS, Motti C, Mattioli PL, Pujia A (1997). In vivo association between low wall shear stress and plaque in subjects with asymmetrical carotid atherosclerosis. Stroke.

[8] Ku DN, Giddens DP, Zarins CK, Glagov S (1985). Pulsatile flow and atherosclerosis in the human carotid bifurcation. Positive correlation between plaque location and low oscillating shear stress. Arteriosclerosis.

[9] Van Popele NM, Grobbee DE, Bots ML, Asmar R, Topouchian J, Reneman RS, Hoeks AP, van der Kuip DA, Hofman A, Witteman JC (2001). Association between arterial stiffness and atherosclerosis: the Rotterdam Study. Stroke.

[10] Harloff A, Strecker C, Reinhard M, Kollum M, Handke M, Olschewski M, Weiller C, Hetzel A (2006). Combined measurement of carotid stiffness and intima-media thickness improves prediction of complex aortic plaques in patients with ischemic stroke. Stroke.

[11] Bots ML, Hofman A, Grobbee DE (1997). Increased common carotid intima-media thickness: adaptive response or a reflection of atherosclerosis?: findings from the Rotterdam Study. Stroke.

[12] Paini A, Boutouyrie P, Calvet D, Tropeano AI, Laloux B, Laurent S (2006). Carotid and aortic stiffness: determinants of discrepancies. Hypertension.

[13] Chobanian AV, Bakris GL, Black HR, Cushman WC, Green LA, Izzo JL, Jones DW, Materson BJ, Oparil S, Wright JT, Roccella EJ, National Heart, Lung, and Blood Institute Joint National Committee on Prevention, Detection, Evaluation, and Treatment of High Blood Pressure; National High Blood Pressure Education Program Coordinating Committee (2003). The Seventh Report of the Joint National Committee on Prevention, Detection, Evaluation, and Treatment of High Blood Pressure: the JNC 7 report. JAMA.

[14] Vermeersch SJ, Rietzschel ER, De Buyzere ML, Van Bortel LM, D’Asseler Y, Gillebert TC, Verdonck PR, Segers P (2007). Validation of a new automated IMT measurement algorithm. J Hum Hypertens.

[15] Barth JD (2002). An update on carotid ultrasound measurement of intima-media thickness. Am J Cardiol.

[16] Gonzalez-Juanatey C, Llorca J, Testa A, Revuelta J, Garcia-Porrua C, Gonzalez-Gay MA (2003). Increased prevalence of severe subclinical atherosclerotic findings in long-term treated rheumatoid arthritis patients without clinically evident atherosclerotic disease. Medicine (Baltimore).

[17] Gnasso A, Carallo C, Irace C, Spagnuolo V, De Novara G, Mattioli PL, Pujia A (1996). Association between intima-media thickness and wall shear stress in common carotid arteries in healthy male subjects. Circulation.

[18] Lee MY, Wu CM, Yu KH, Chu CS, Lee KT, Sheu SH, Lai WT (2009). Association between wall shear stress and carotid atherosclerosis in patients with never treated essential hypertension. Am J Hypertens.

[19] Samijo SK, Willigers JM, Brands PJ, Barkhuysen R, Reneman RS, Kitslaar PJ, Hoeks AP (1997). Reproducibility of shear rate and shear stress assessment by means of ultrasound in the common carotid artery of young human males and females. Ultrasound Med Biol.

[20] Lee JH, Cho KI, Kim SM (2012). Carotid arterial stiffness in patients with rheumatoid arthritis assessed by speckle tracking strain imaging: its association with carotid atherosclerosis. Clin Exp Rheumatol.

[21] Malek AM, Alper SL, Izumo S (1999). Hemodynamic shear stress and its role in atherosclerosis. JAMA.

[22] Koller A, Huang A (1999). Development of nitric oxide and prostaglandin mediation of shear stress-induced arteriolar dilation with aging and hypertension. Hypertension.

[23] Chatzizisis YS, Coskun AU, Jonas M, Edelman ER, Feldman CL, Stone PH (2007). Role of endothelial shear stress in the natural history of coronary atherosclerosis and vascular remodeling: molecular, cellular, and vascular behavior. J Am Coll Cardiol.

[24] Eigenbrodt ML, Bursac Z, Rose KM, Couper DJ, Tracy RE, Evans GW, Brancati FL, Mehta JL (2006). Common carotid arterial interadventitial distance (diameter) as an indicator of the damaging effects of age and atherosclerosis, a cross-sectional study of the Atherosclerosis Risk in Community Cohort Limited Access Data (ARICLAD), 1987–89. Cardiovasc Ultrasound.

[25] Safar ME, London GM, Asmar R, Frohlich ED (1998). Recent advances on large arteries in hypertension. Hypertension.

[26] Boutouyrie P, Bussy C, Lacolley P, Girerd X, Laloux B, Laurent S (1999). Association between local pulse pressure, mean blood pressure, and large-artery remodeling. Circulation.

[27] Chironi G, Gariepy J, Denarie N, Balice M, Megnien JL, Levenson J, Simon A (2003). Influence of hypertension on early carotid artery remodeling. Arterioscler Thromb Vasc Biol.

[28] Jiang YN, Kohara K, Hiwada K (1998). Alteration of carotid circulation in essential hypertensive patients with left ventricular hypertrophy. J Hum Hypertens.

[29] Kohara K, Jiang Y, Igase M, Hiwada K (1999). Effect of reflection of arterial pressure on carotid circulation in essential hypertension. Am J Hypertens.

[30] Mattace-Raso FU, van der Cammen TJ, Hofman A, van Popele NM, Bos ML, Schalekamp MA, Asmar R, Reneman RS, Hoeks AP, Breteler MM, Witteman JC (2006). Arterial stiffness and risk of coronary heart disease and stroke: the Rotterdam Study. Circulation.

[31] Laurent S, Boutouyrie P (2007). Arterial stiffness: a new surrogate end point for cardiovascular disease?. J Nephrol.

[32] Traub O, Berk BC (1998). Laminar shear stress: mechanisms by which endothelial cells transduce an atheroprotective force. Arterioscler Thromb Vasc Biol.

[33] Carallo C, Lucca LF, Ciamei M, Tucci S, de Franceschi MS (2006). Wall shear stress is lower in the carotid artery responsible for a unilateral ischemic stroke. Atherosclerosis.

[34] Tuka V, Slavikova M, Svobodova J, Malik J (2006). Diabetes and distal access location are associated with higher wall shear rate in feeding artery of PTFE grafts. Nephrol Dial Transplant.

[35] Setty SP, Salles-Cunha S, Scissons R, Begeman G, Farison J, Beebe HG (2002). Noninvasive measurement of shear rate in autologous and prosthetic bypass grafts. Vasc Endovasc Surg.

[36] Paszkowiak JJ, Dardik A (2003). Arterial wall shear stress: observations from the bench to the bedside. Vasc Endovasc Surg.

