# Supplementary figures and images for: Wall shear stress in hypertensive patients is associated with carotid vascular deformation assessed by speckle tracking strain imaging
Source: Clin Hypertens. 2014 Sep 25;20:10. doi: 10.1186/2056-5909-20-10 (PMC4750791; doi:10.1186/2056-5909-20-10)

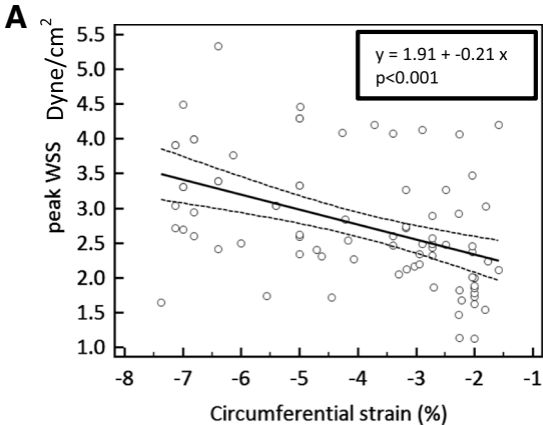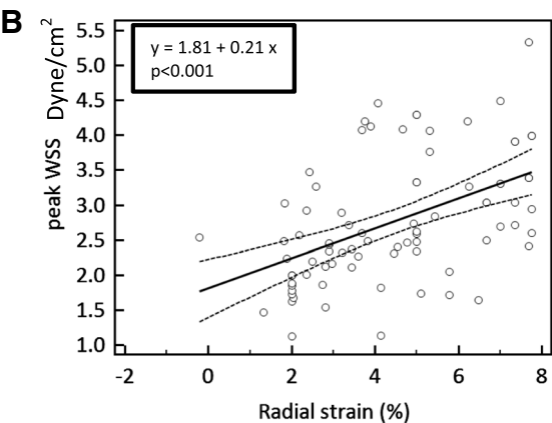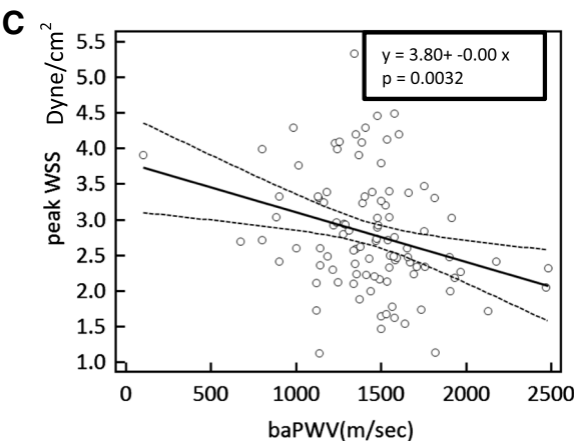

Supplement: Supplementary file 1 — Authors’ original file for figure 1 [file 40885_2014_10_MOESM1_ESM.pdf]
